# Genome-wide mapping of gene-microbe interactions in the murine lung microbiota based on quantitative microbial profiling

**DOI:** 10.1186/s42523-023-00250-y

**Published:** 2023-06-01

**Authors:** C. J. Chung, B. M. Hermes, Y. Gupta, S. Ibrahim, Meriem Belheouane, John F. Baines

**Affiliations:** 1 Max Planck Institute for Evolutionary Biology, August-Thienemann-Str. 2, 24306 Plön, Germany; 2 Section of Evolutionary Medicine, Institute for Experimental Medicine, Kiel University, Arnold-Heller-Str. 3, 24105 Kiel, Germany; 3 Division of Nephrology, Department of Medicine, Columbia University Irving Medical Center, New York, NY 10032 USA; 4 College of Medicine and Health Sciences, Khalifa University, Abu Dhabi, UAE; 5 Research Center Borstel, Evolution of the Resistome, Leibniz Lung Center, Parkallee 1-40, 23845 Borstel, Germany

**Keywords:** Mouse, Lung microbiota, Host genetics, QTL mapping, Quantitative microbial profiling, *Lactobacillus*, *Interleukin 10*

## Abstract

**Background:**

Mammalian lungs comprise a complex microbial ecosystem that interacts with host physiology. Previous research demonstrates that the environment significantly contributes to bacterial community structure in the upper and lower respiratory tract. However, the influence of host genetics on the makeup of lung microbiota remains ambiguous, largely due to technical difficulties related to sampling, as well as challenges inherent to investigating low biomass communities. Thus, innovative approaches are warranted to clarify host-microbe interactions in the mammalian lung.

**Results:**

Here, we aimed to characterize host genomic regions associated with lung bacterial traits in an advanced intercross mouse line (AIL). By performing quantitative microbial profiling (QMP) using the highly precise method of droplet digital PCR (ddPCR), we refined 16S rRNA gene amplicon-based traits to identify and map candidate lung-resident taxa using a QTL mapping approach. In addition, the two abundant core taxa *Lactobacillus* and *Pelomonas* were chosen for independent microbial phenotyping using genus-specific primers. In total, this revealed seven significant loci involving eight bacterial traits. The narrow confidence intervals afforded by the AIL population allowed us to identify several promising candidate genes related to immune and inflammatory responses, cell apoptosis, DNA repair, and lung functioning and disease susceptibility. Interestingly, one genomic region associated with *Lactobacillus* abundance contains the well-known anti-inflammatory cytokine *Il10*, which we confirmed through the analysis of *Il10* knockout mice.

**Conclusions:**

Our study provides the first evidence for a role of host genetic variation contributing to variation in the lung microbiota. This was in large part made possible through the careful curation of 16S rRNA gene amplicon data and the incorporation of a QMP-based methods. This approach to evaluating the low biomass lung environment opens new avenues for advancing lung microbiome research using animal models.

**Graphical Abstract:**

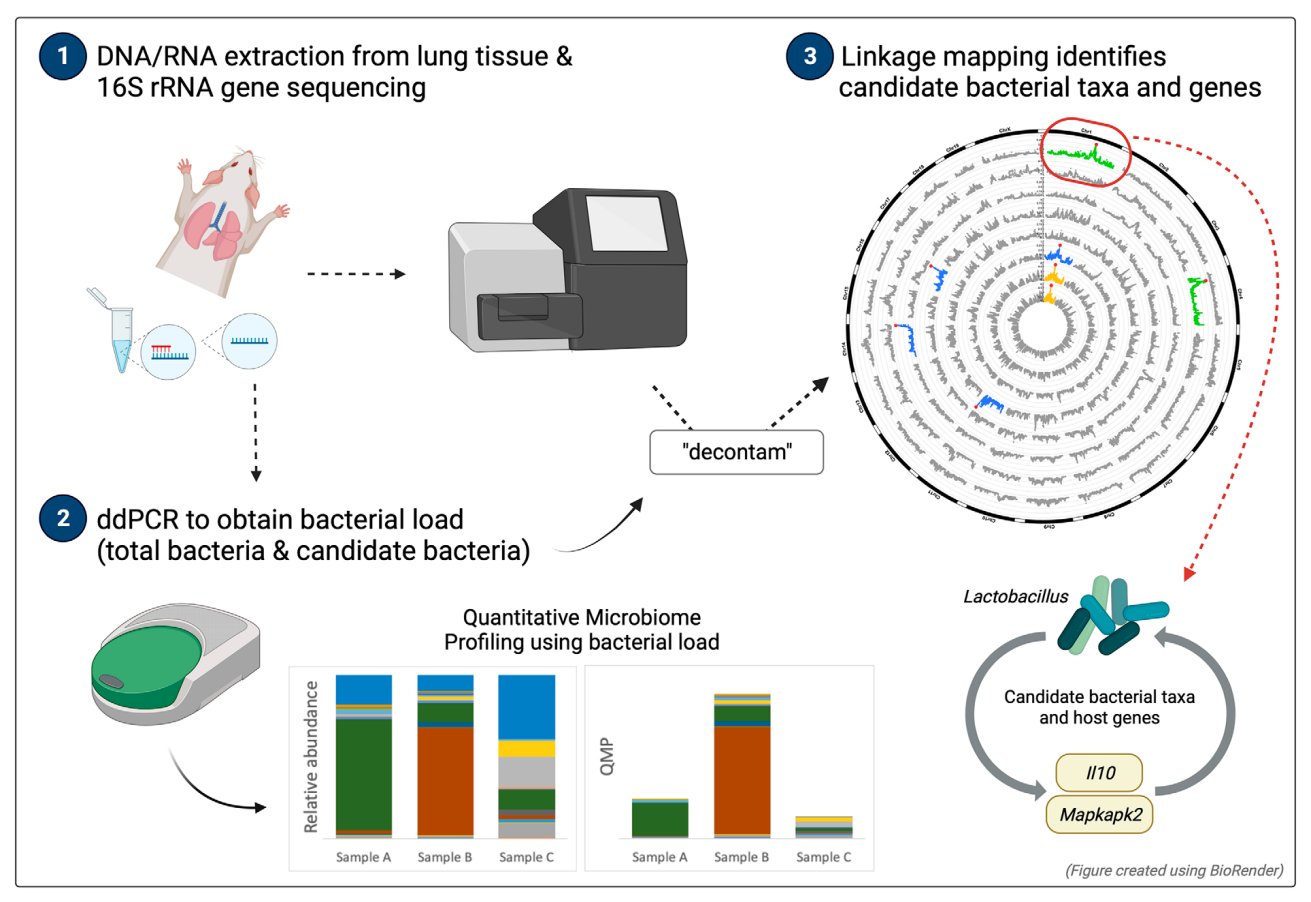

**Supplementary Information:**

The online version contains supplementary material available at 10.1186/s42523-023-00250-y.

## Background

Healthy lungs house a diverse and complex microbial ecosystem that contributes to critical aspects of host biology. Previous surveys of lung microbiota reveal microbial alterations in the context of disease, including cystic fibrosis [1], asthma [2], chronic obstructive pulmonary disease [3, 4], lung cancer [5, 6], as well as COVID-19 [7–9]. Thus, revealing the fundamental forces that govern the assembly and stability of bacterial communities in the lung is of critical importance for understanding its role in health and disease. Environmental factors, including smoking status [3, 10], infant feeding mode [11], early life stress [12], household [13, 14], and antibiotic use [15, 16] are identified as significant contributors to upper and lower respiratory tract bacterial community structure. However, there is a paucity of research exploring the influence of host genetics on the makeup of lung microbiota.

The role of host genetics on bacterial community structure at other body sites has been demonstrated using twin designs [17], comparison of mouse inbred strains [18, 19], genome-wide association studies [20–24], and quantitative trait locus (QTL) analyses [25, 26]. These studies are largely limited to the gut, although researchers are beginning to successfully apply these methods to low-biomass communities. For example, we previously used a novel QTL mapping approach in a murine skin microbiota study whereby we extended bacterial trait mapping to both 16S rRNA gene copy (DNA) as well as 16S rRNA gene transcripts (RNA) [27]. Microbial profiling based on RNA as template preferentially reflects living/active cells, and accordingly increased the number of significant associations detected between the host and resident skin microbes [27].

Microbiome research on the human lung environment still lags, largely due to technical sampling challenges unique to this site [28–32]. Bronchoalveolar lavage (BAL), the established best practice for sampling human lung microbiota, is invasive, costly, requires sedation, and poses unnecessary risks to healthy subjects [31]. These barriers impede sampling large numbers of individuals, which is required to detect biological signals with methods such as GWAS [31]. Non-invasive sampling strategies, such as sputum or tracheal aspirate collection, are more accessible, but complicated by poorly defined methods for sputa processing, ambiguous origins of collected microbiota, and a substantial risk of contamination from the oropharynx [31, 33]. Moreover, the low biomass of bacterial samples collected from the lower airways poses further challenges for handling contamination. The so-called “kitome” of nucleic acid extraction kits and reagents, as well as laboratory environments, are well-documented sources of contamination that can radically affect data interpretation, as contaminants tend to be preferentially amplified and sequenced over true microbial signal in low biomass samples [34–40]. Thus, novel approaches that can be readily translated to human research are needed to advance our understanding of dynamic host-microbe interactions in the mammalian lung.

In this study, we aimed to improve the experimental profiling of resident lung microbes for QTL mapping of the lung microbiota, using a mouse advanced intercross line (AIL) that was previously successful for genetic mapping in both the gut [41] and low biomass environment of the skin [27, 42]. For this, we employed a strategy to first screen for taxa that are likely to be true lung residents using 16S rRNA gene amplicon profiles at the transcript (RNA) level, followed by measurements of overall bacterial load and selected individual taxa using the highly precise method of droplet digital PCR (ddPCR) (Graphical abstract). QTL linkage mapping of lung microbiota using ddPCR-based estimates revealed significant associations with host loci, whose confidence intervals contain genes related to immune and inflammatory responses, cell apoptosis, and DNA repair. Further, a significant association between *Lactobacillus* abundance and a region of the mouse genome containing *Il10*, a well-known anti-inflammatory cytokine, was confirmed through the analysis of *Il10* knockout mice. These data suggest that incorporating quantitative profiling from ddPCR bacterial load measurements for use in linkage mapping may improve study reliability, and thus open new avenues for advancing lung microbiome research.

## Results

### AIL mouse population and overall study design

We analyzed 242 lung tissue samples derived from the 15th generation of a previously established AIL population, as described by Belheouane et al. [27]. In brief, the AIL consisted of MRL/MpJ, NZM2410/J, BXD2/TyJ, and CAST/EiJ mice (Jackson Lab, Maine, USA). To create a heterogenous intercross line, mice were intercrossed in equal strain and sex distributions [27, 42].

To map genomic regions associated with bacterial traits in the murine lung, we carried out a nested strategy to identify and map candidate resident taxa while minimizing the influence of potential contamination (see Methods). First, we screened for putative bacterial lung residents by analyzing 16S rRNA gene amplicon profiles at the transcript (RNA) level, thereby preferentially identifying live/active taxa. These data were further curated through the application of the “decontam” R package [34], which incorporates information from negative controls and absolute quantification of bacterial load to identify possible contaminants in metagenomic sequencing data (see Methods). For this purpose, we applied ddPCR to obtain precise total bacterial load measurements. Next, a core measurable microbiota (CMM) was defined based on the processed sequences derived from RNA template for further analysis. In a second step, we measured the bacterial loads of individual candidate taxa using ddPCR. Finally, linkage mapping was performed on the following panel of traits: (1) CMM based on conventional relative abundance estimates, derived from RNA template (herein: CMM-RA), (2) CMM based on relative abundance corrected by quantitative microbiome profiling (QMP; i.e., ddPCR estimates of bacterial load), derived from RNA template (herein: CMM-QMP), (3) ddPCR estimates of candidate taxa derived from DNA template (herein: ddPCR-DNA), and (4) ddPCR estimates of candidate taxa derived from RNA template (herein: ddPCR-RNA).

### 16S rRNA gene sequencing and ddPCR to define bacterial traits

**Fig. 1:**
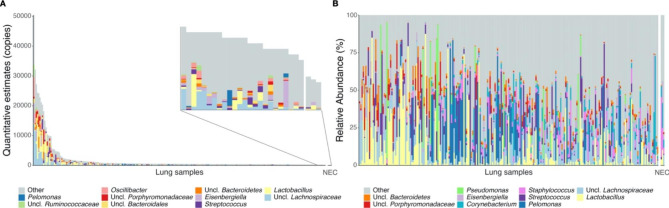
Lung bacterial community composition of AIL mice. Samples are ordered according to bacterial load in both panels, with the taxonomy displayed for the ten most abundant genera, determined separately, for **(A)** QMP and **(B)** relative abundance data. The lowest 10% of the samples based on bacterial load is shown in the zoomed window of panel A, for which the three negative extraction controls (NEC) are the lowest

To identify resident candidate taxa from the lung, we first performed 16S rRNA gene amplicon sequencing on both DNA and RNA reverse transcribed into complementary DNA (cDNA) as template (see Methods). After sequence processing, we determined the DNA-based data to be of insufficient quality/quantity, similar to another report that attempted to sequence DNA from murine lung samples using a comparable PCR protocol ([43]; note other studies using a two-step PCR protocol/higher number cycles yielded different results, e.g. [44–47]). However, rather than adopting a two-step PCR protocol as in Barfod et al. [43], which may be more prone to amplifying contaminants in low biomass samples [48], we instead narrowed the 16S sequencing data analysis to the transcript (RNA) level. This reflects metabolically active cells [49, 50], and may better reveal true resident lung bacteria interacting with the host, as demonstrated in our QTL mapping study exploring gene-microbe interactions in the skin of this same AIL [27]. To assess for potential contamination, we employed the “frequency” method from the “decontam” R package (v.1.4.0) using a threshold of 0.1 (see Methods). This analysis resulted in the removal of potential contaminant amplicon sequence variants (ASVs), including the common contaminants *Halomonas* and *Shewanella*. In total, we analyzed 8,414,939 sequences, and after normalizing sequencing coverage to 4,000 sequences per sample, a total of 20,772 ASVs remained in the data set. We first analyzed murine lung bacterial community composition at the genus and ASV levels based on 16S rRNA gene profiles (Fig. 1, Additional file 1: Figure S1). The most abundant genera include *Lactobacillus*, uncl. *Lachnospiraceae, and Pelomonas*, with a mean relative abundance of 9.85, 8.79, and 7.50%, respectively (Additional file 2: Table S1). *Lactobacillus* and *Pelomonas* were additionally selected as bacterial candidate traits for targeted linkage mapping using ddPCR (see below).

Next, we defined a CMM [25, 26] (see Methods) consisting of 58 taxa ranging from the genus- to phylum-level, as well as 13 ASVs. The number of CMM traits represents a small fraction of the total number of lung microbiota (e.g., 1.74% of genera and 0.06% of ASVs), but their abundances represent 55.16% and 22.38% of the respective taxa at these levels.

Because 16S rRNA gene amplicon profiling is prone to biases and errors related to relative abundance compositional analysis [51], we performed QMP using ddPCR-based bacterial load estimates at the RNA level to account for these concerns. 16S rRNA sequence-derived relative abundances of the CMM traits were then subsequently transformed into corrected, absolute quantitative abundances [51] (see Methods), i.e., CMM-QMP traits. Thereafter, the quantitative profiles of the 58 taxa and 13 ASVs were included as an independent set of bacterial traits.

Finally, as indicated above, we selected two candidate bacterial traits, *Lactobacillus* and *Pelomonas*, for further independent analysis, as they were the two most abundant classifiable genera and are frequently identified as lung residents [43, 52, 53]. Droplet digital PCR is ideal for quantifying low biomass samples, as the process of fractionating a sample into thousands of individual droplets, in which independent PCR reactions occur, allows for the amplification of even very low levels of target strains [4, 54–56]. This was demonstrated by e.g., Gobert et al., who effectively measured low levels of *Lactobacillus* in fecal samples using ddPCR [54]. We thus generated load estimates for *Lactobacillus* and *Pelomonas* using genus-specific primers adapted for ddPCR (see Methods). This was performed at both the DNA and RNA level, as (i) ddPCR is sensitive enough to allow for absolute quantification and (ii) taxon-specific primers are expected to be less prone to contaminating taxa than universal PCR primers. Importantly, these specific estimates are significantly correlated to those based on 16S rRNA gene sequencing for both *Lactobacillus* (ddPCR-RNA vs. CMM-RA: Spearman’s r = 0.5848, *p* < 2.2 × 10^− 16^; ddPCR-RNA vs. CMM-QMP: r = 0.7131, *p* < 2.2 × 10^− 16^) and *Pelomonas* (ddPCR-RNA vs. CMM-RA: r = 0.2519, *p* = 7.411 × 10^− 05^; ddPCR-RNA vs. CMM-QMP: r = 0.2916, *p* = 3.796 × 10^− 06^), whereby the correlation is stronger for the CMM-QMP traits.

### Summary statistics and evaluation of sources of variation for lung bacterial traits

Prior to genetic mapping, it is important to determine whether (i) a given phenotype displays sufficient variation between individuals, and (ii) any residual variation remains after accounting for potential covariates such as cage, sex, or age. Summary statistics for each of the four categories of bacterial traits are provided in Additional file 2: Tables S1-3. This reveals considerable interindividual variation among traits. In particular, traits with large mean abundances, such as *Lactobacillus* and *Pelomonas*, display a large range, from 0 to 88.15% and 0-82.85% across the dataset for the CMM-RA measurements (Additional file 2: Tables S1-3).

Next, we evaluated the influence of host and environmental factors on bacterial trait variation in each of the four categories by constructing a mixed effects model that includes sex and age as fixed explanatory variables, cage as a random effect, and the bacterial trait values as responses (see Methods; Additional file 2: Table S4-6). These variables are associated with variation in trait values to varying degrees. For example, cage explains 0% of the total variance for *Lactobacillus*, but explains 28.83% of the total variance for *Pelomonas* among CMM-RA traits. However, importantly, the remaining residual variation in all four categories of traits accounts for the greatest proportion of total variance after accounting for sex, age, and cage effects. This suggests that other variables differing between individual mouse hosts, such as genotype, may contribute to variation in bacterial trait abundances.

### QTL mapping of lung microbiota traits

**Fig. 2:**
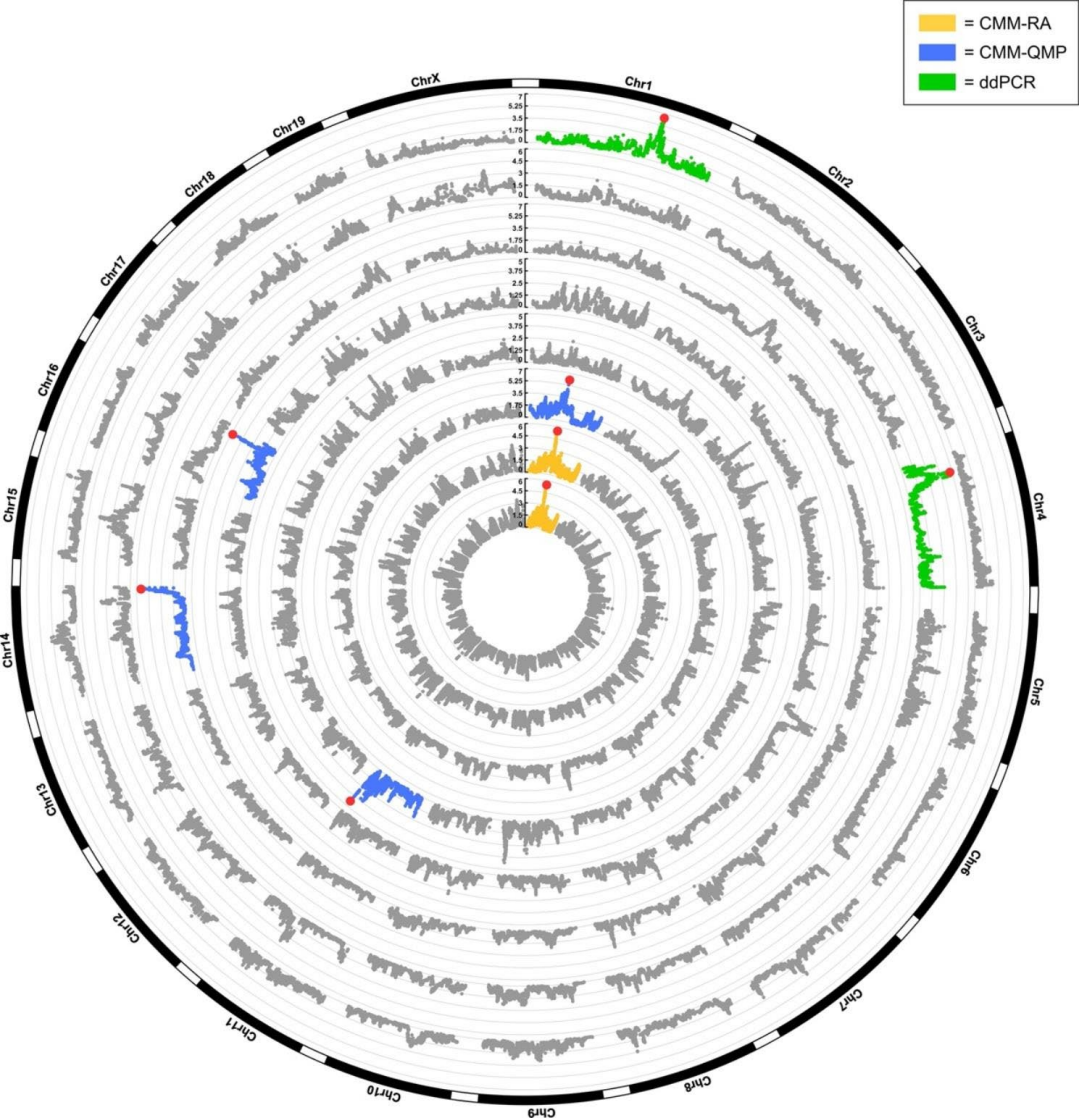
Manhattan plots from linkage mapping of bacterial traits. From inner to outer circles: *Enterobacteriales* CMM-RA, *Enterobacteriaceae* CMM-RA, *Deltaproteobacteria* CMM-QMP, *Pelomonas* CMM-QMP, *Streptococcus* CMM-QMP, ASV8_*Propionibacterium* CMM-QMP, *Pelomonas* ddPCR-RNA, and *Lactobacillus* ddPCR-DNA. CMM-RA traits are shown in yellow, CMM-QMP in blue, and ddPCR-DNA/RNA in green. Peak SNPs are highlighted with red dots. LOD score on the y-axis indicates the -log *p* value of the association between a locus and a phenotypic trait

To test for associations between resident lung bacteria and the host genome, we performed QTL mapping for the CMM-RA and CMM-QMP traits, as well as for the ddPCR-DNA and -RNA for *Lactobacillus* and *Pelomonas*. In total, this yielded seven significant (*p* ≤ 0.05) and six suggestive (*p* ≤ 0.1) host loci involving eight bacterial traits (CMM-RA: one significant, five suggestive; CMM-QMP: four significant, one suggestive; ddPCR-DNA/RNA: two significant), with narrow confidence intervals ranging from 0.08 to 3.59 Mb, with an average of 1.80 Mb (Table 1, Additional file 3: Table S7). The number of protein-coding genes within these confidence intervals ranged from one to 36. For example, two significant associations were detected among the CMM-QMP traits at the genus-level, including *Pelomonas* and *Streptococcus* at chromosomes 11 and 16, for which the confidence intervals contained one and eight genes, respectively. For ddPCR-DNA and -RNA, *Lactobacillus* and *Pelomonas* were each significantly (*p* < 0.05) associated with a single genomic locus (Fig. 2; Table 1).

**Table 1:** QTL mapping statistics for CMM-RA, CMM-QMP, and ddPCR-DNA/RNA with significant associations

Type	Taxon	Trait	Chr	Peak SNP	LOD score	Confidence interval	Size (Mb)	Phenotypic variance (%) assoc. with peak SNP
CMM-RA	Order	*Enterobacteriales*	1	UNC1088683	5.49	88.61–90.40	1.79	9.92
	Family	*Enterobacteriaceae*	1	UNC1088683	5.49	88.61–90.40	1.79	9.92
CMM-QMP	Class	*Deltaproteobacteria*	1	UNC1143014	6.03	93.30–95.02	1.72	11.01
	Genus	*Pelomonas*	11	UNC20541010	4.42	121.68–121.76	0.08	8.19
		*Streptococcus*	16	JAX00427186	4.85	86.88–88.15	1.27	8.96
	ASV	ASV8_*Propionibacterium*	14	UNC24933570	6.36	122.04–125.01	2.97	11.58
ddPCR-DNA		*Lactobacillus*	1	UNC1677482	6.38	131.79–133.44	1.65	11.43
ddPCR-RNA		*Pelomonas*	4	UNC6891976	5.74	21.98–25.57	3.59	10.35

**Fig. 3:**
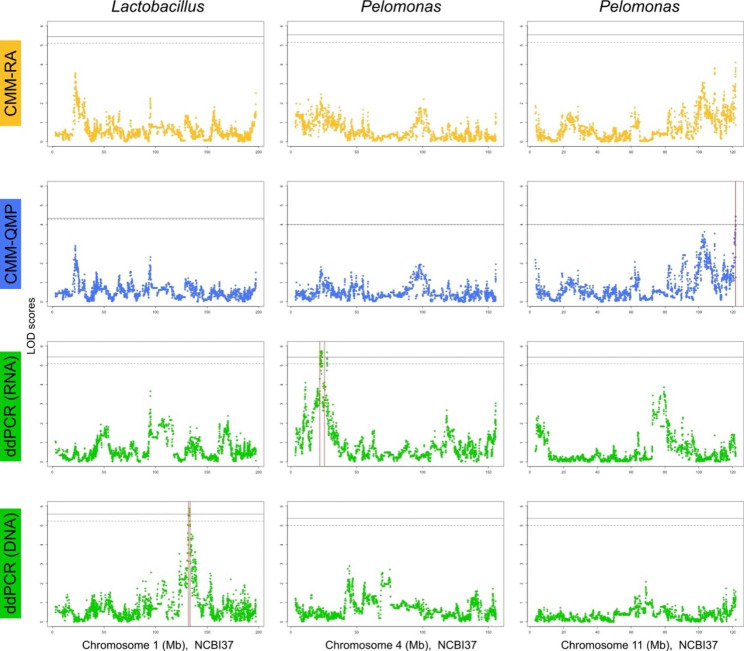
Overview and comparison of the Manhattan plots of *Lactobacillus* and *Pelomonas* traits from CMM-RA, CMM-QMP, and ddPCR-DNA and ddPCR-RNA, with each column dedicated to the chromosome where the QTL was detected; *Lactobacillus* ddPCR-DNA at chromosome 1, *Pelomonas* ddPCR-RNA at chromosome 4, and *Pelomonas* CMM-QMP at chromosome 11, from left to right. Solid black lines indicate significant thresholds and dashed lines indicate suggestive thresholds of each trait. Red vertical lines indicate confidence intervals of the QTL.

Overall, the phenotyping performed for the two abundant core taxa *Lactobacillus* and *Pelomonas* was based on four different methods, which differ according to (i) primers (universal vs. taxon-specific), (ii) relative abundance vs. quantitative estimates, and (iii) DNA vs. RNA. To compare these methods, we generated four respective QTL profile plots for each of the three significant associations involving these two taxa (Fig. 3). For the *Lactobacillus* association on chromosome 1, only the ddPCR-DNA measurements revealed a significant association, which suggests that this region of the genome associates only to their cell number rather than their activity. In contrast, the *Pelomonas* QTL on chromosome 4 involves only the ddPCR-RNA estimates. Lastly, the *Pelomonas* QTL on chromosome 11 involves only the CMM-QMP estimates. A similar peak structure is apparent for the CMM-RA estimate, but does not reach the significant or suggestive threshold. Thus, with regard to the three categories mentioned above, the involvement of quantitative- over relative abundance information appears to be most important for the detection of gene-microbe interactions, while differences between primers and/or cell number vs. activity may lead to discrepancies between phenotyping methods.

### Analysis of candidate genomic regions

The narrow confidence intervals afforded by the AIL population allowed us to identify several promising candidate genes. In Table 2, we report a list of candidate genes from significant QTLs and their functions, summarized from experimental evidence. Most genes within the candidate regions are related to immune response, inflammatory response, cell apoptosis, and/or DNA repair. A number of genes are notable due to their role in lung functioning and disease susceptibility. For the QTL on chromosome 1 associated with variation in *Lactobacillus* ddPCR-DNA, the *Mk2* (mitogen-activated protein kinase-activated protein kinase 2) and the *Il10* (*Interleukin 10*) genes are the two closest genes in proximity to peak SNP UNC1677482. In humans, MK2 is a downstream product of the p38^MAPK^ pathway acting as a pro-inflammatory kinase, and induces various signals such as cytokines in response to lipopolysaccharide (LPS)- and virus-induced infections [58–61]. MK2 is involved in transcriptional and post-transcriptional regulation of cytokine expression and was previously shown to affect the stability of *Il10* transcript [59]. IL-10, a well-known anti-inflammatory cytokine, is e.g., also involved in mitigating disease severity in *Mycobacterium tuberculosi*s infection [62]. For the *Pelomonas* (ddPCR-RNA) QTL on chromosome 4, the peak SNP lies within an intergenic region. However, several genes are found within the confidence interval, including *Pou3f2* and *Mms22l*. POU family transcription factors were previously shown to be highly expressed in small-cell lung carcinoma (SCLC) cell lines and to contribute to neuroendorcrine differentiation in non-small cell lung carcinoma (NSCLC) cell lines[63]. *MMS22L* was shown to accelerate cancer cell growth in lung cancer cell lines [64]. In contrast to the other candidate genes, *Klhl32* is poorly characterized. However, recent work by de Vries et al. [65] identified *KLHL32* as a protein-coding gene that strongly associates with DNA methylation levels of a specific CpG-site (a cytosine base located adjacent to a guanine base) in patients with chronic obstructive pulmonary disease (COPD).

**Table 2:** List of candidate genes within confidence intervals and their functions

	Trait	Gene	Functions (& references)
**RA**	*Enterobacteriaceae* / *Enterobacteriales*	*Chrnd*, *Chrng*	Nicotine dependence, smoking behaviors, lung cancer, COPD [66]; lung hypoplasia [67]
		*Ecel1*	Restrictive lung disease (affected by respiratory muscles) [68]
		*Dis3l2*	Lung function [69]
		*Kcnj13*	Development & physiology of the respiratory system [70]; tracheal tubulogenesis [71]
**QMP**	*Deltaprotebacteria*	*Per2*	NSCLC [72, 73]; lung tumorigenesis [74]
		*Twist2*	Lung cancer [75]; lung adenocarcinoma [76]; Pneumonia [77]
	*Pelomonas*	*Ptchd3*	Asthma [78]
	*Streptococcus*	*Grik1*	Lung metastasis (of colorectal cancer in vivo) [79]; lung cancer [80]
		*Bach1*	Lung cancer [81–83]; cystic fibrosis [84]
		*Map3k7cl*	NSCLC [85]; pulmonary cell development [86]
	ASV8_*Propionibacterium*	*Nalcn*	Respiratory rhythm [87]; NSCLC [88]
		*Fgf14*	Lung Adenocarcinomas [89, 90]; lung functioning & phenotype [91]
		*Ubac2*	COPD [92]; cystic fibrosis [93]; asthma [94]
		*Zic2*	Lung adenocarcinoma [95]; NSCLC [96]; SCLC [97, 98]
**ddPCR-DNA**	*Lactobacillus*	*Mk2*	Lung cancer [99]; inflammatory pulmonary diseases [100]
		*Il10*	Tuberculosis [101]; asthma [102]; NSCLC [103, 104]
		*Il19*	Antimicrobial defense in airway epithelial cells [105]
		*pIgR*	COPD-like phenotype airway inflammation [106]
**ddPCR-RNA**	*Pelomonas*	*Pou3f2*	NSCLC [63]
		*Mms22l*	Lung carcinogenesis [64]
		*Klhl32*	COPD [65]
		*Fut9*	Bronchopulmonary dysplasia [107]

Key: COPD: chronic obstructive pulmonary disorder, NSCLC: non-small cell lung cancer, SCLC: small cell lung cancer

### Evaluation of ***Lactobacillus*** in a ***Il10*** knockout model

**Fig. 4:**
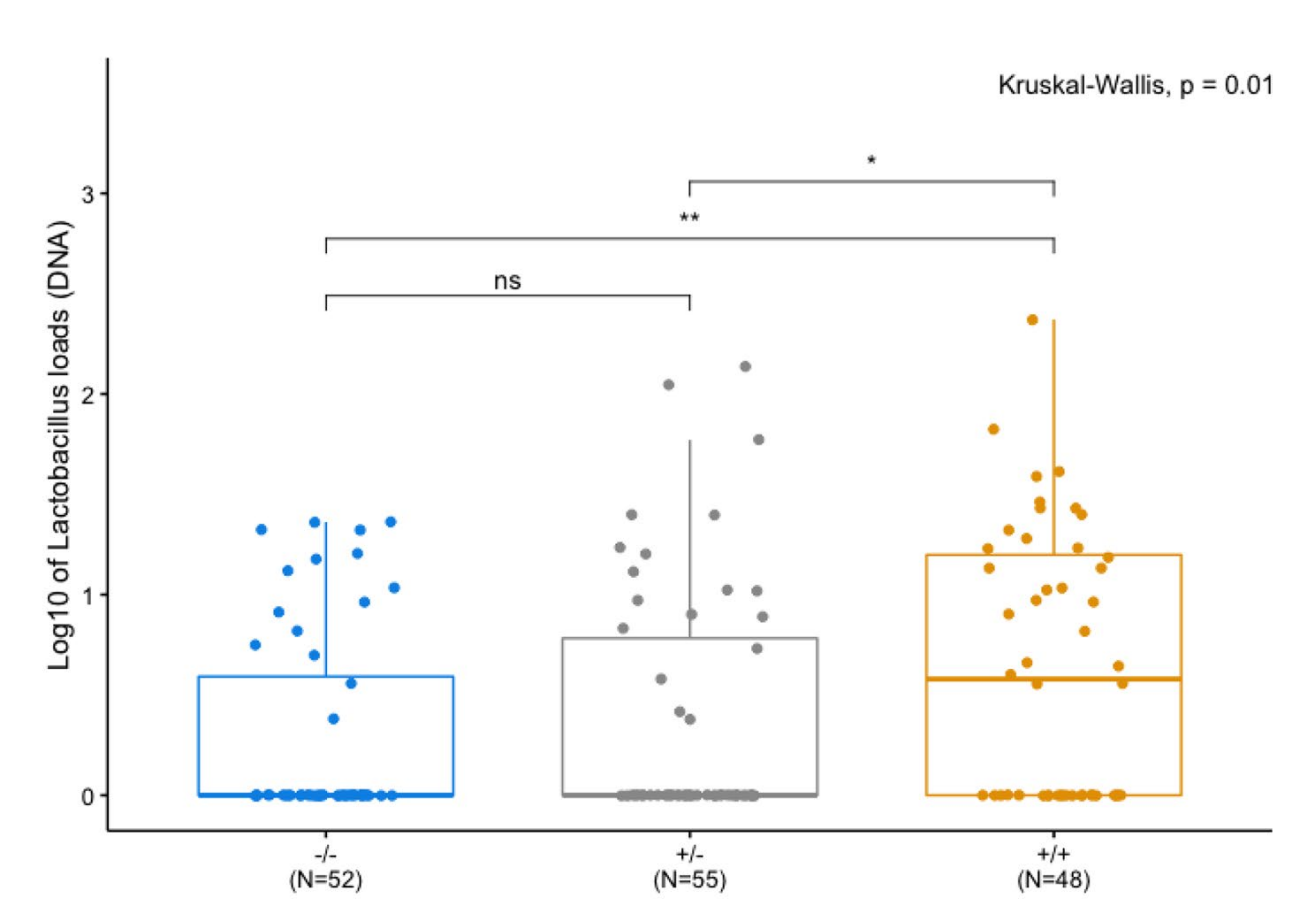
*Lactobacillus* load according to *Il10* genotype. Bacterial loads determined by ddPCR (DNA) were log_10_-transformed and compared using Kruskal-Wallis tests with Wilcoxon signed-rank post-hoc tests. *P* values were corrected for multiple testing according to Benjamini and Hochberg [108]. Error bars depict standard error. *p* < 0.01 **, *p* < 0.05 *, not significant “ns”

Given the promising association detected between *Lactobacillus* load (DNA) and a locus containing the well-known anti-inflammatory cytokine IL-10, we aimed to confirm this potential gene-microbe association in an *Il10* knockout model using the same phenotyping method as for our mapping population. We accordingly performed ddPCR to quantify the *Lactobacillus* load at the DNA level using genus-specific primers in *Il10*^*+/+*^, *Il10*^*+/−*^ and *Il10*^*−/−*^ mice. Interestingly, we observe significant differences in *Lactobacillus* according to genotype, with *Il10*^+/+^ mice displaying higher abundances than *Il10*^*+/−*^ and *Il10*^*−/−*^ mice (Fig. 4). We confirmed that the loads were associated only with genotype and not with any other variables including sex, cage, age, and cross (Kruskal Wallis; p > 0.05).

## Discussion

In this study, we applied a rigorous combination of experimental- and data curation procedures that enabled us to carry out the first systematic assessment of host genetic effects on the mammalian lung microbiota. From the panel of traits defined for mapping, we identified seven significant and six suggestive host gene-microbial associations. Importantly, we found that incorporating quantitative bacterial load estimates in defining microbial phenotypes to be more effective in identifying gene-microbe associations than 16S rRNA gene amplicon profiles alone. While we identified only two significant associations among CMM-RA traits, five further significant associations were revealed after incorporating bacterial load information (i.e., CMM-QMP and ddPCR). Previous studies of the lung microbiota have identified two of the bacterial taxa involved, *Lactobacillus* [32, 53, 109] and *Pelomonas* [43], and these may represent key inhabitants of this host habitat. Moreover, we confirmed an association between *Lactobacillus* and a genomic region containing the *Il10* gene in an independent *Il10* knockout mouse model. This confirmation is of particular significance given the expected impact of environmental differences on the lung microbiota, as the *Il10* mice were housed and analyzed in a completely separate facility with differences in food, bedding, caretakers, etc.

*Lactobacillus* are known to inhabit the mammalian lung and are generally regarded as probiotic bacteria [52]. Additionally, previous research showed *Lactobacillus* to modulate host immune responses and to reduce injury during lung infection [110–112]. Our targeted mapping approach revealed *Lactobacillus* load to be associated with a region containing the important candidate genes *Il10* and *Mk2*. The functional relationship between *Lactobacillus* and these genes remains unclear, although a number of studies suggest a clinically relevant link. A previous study administered the *Lactobacillus casei* strain Shirota (LcS) to 2-week-old mice, which were then challenged with ovalbumin to induce allergic symptoms in the lungs [113]. This revealed modified immune response, including increased IL-10 levels, in mice administered LcS compared to controls. Although it seems that the effect is dependent on species or strain [114, 115], research continues to demonstrate a beneficial role of *Lactobacillus* in immunity. For example, nasal administration of *L. casei* aided lung recovery from *S. pneumoniae* infection [112], and oral administration of *L. rhamnosus* caused changes in cytokine levels that aided recovery from lung injury and inflammation induced by a synthetic analog of viral double stranded RNA, poly(I:C) [111]. Given this evidence, it is possible that *Lactobacillus* and/or its metabolites modulate the phosphorylation of p38^MAPK^, perhaps through bacterial byproducts or components of the bacterial cell wall [116, 117], which affect downstream signals that support lung function and recovery. Alternatively, *Mk2* gene expression and subsequent IL-10 production may affect the growth or modify the abundance of *Lactobacillus* in the lung.

Likewise, *Pelomonas* was previously identified as a member of the murine lung bacterial community [43]. In humans, recent studies identified *Pelomonas* in the oropharynx of patients infected with SARS-CoV-2 virus [118] and also in breast cancer patients [119], with associations with multiple cytokines and immune genes. Yet, the role of *Pelomonas* in the mammalian lung remains largely unknown. A potential mechanism might be via $\gamma$-aminobutyric acid (GABA) found in the pulmonary neuroendocrine cells (PNEC) distributed along the alveolar airway epithelium [120]. GABA acts as a mediator in mucus production and airway smooth muscle toning and contraction [121–123]. Interestingly, *Pelomonas* was found to contribute to contraction frequency in hydra [124]. Although the potential interplay between *Pelomonas* and GABA in the lungs awaits experimental investigation, we speculate that this may contribute to crosstalk between the host and bacteria. Moreover, given its association with *Pou3f2* and *Mms22l*, *Pelomonas* might play a role in susceptibility to lung cancer and other related lung diseases. Additionally, POU transcription factors are specifically expressed in small cell lung cancer (SCLC), contributing to accelerating cell growth, and POU3F2 was revealed to maintain the proneuronal/neuroendocrine phenotype of SCLC [125]. *MMS22L* was found to be over-expressed in clinical and esophageal cancers, playing a role in growth and survival of cancer cells [64]. This gene might impact the efficacy of DNA-damaging agents, as the knockout of the gene enhances cancer cell apoptosis [64]. Based on these observations, both *Pou3f2* and *Mms22l* might serve as cancer therapy targets, which could be aided by the mechanistic understanding of a possible interaction with *Pelomonas*.

Other QTL intervals also include interesting genes associated with lung or respiratory tract development, functioning, and/or diseases that are potentially supported by the host-microbe interactions. *Enterobacteriaceae* and *Enterobacteriales* were found to be associated with QTL on chromosome 1 with a narrow confidence interval of 1.79 Mb. Within this interval, *Chrnd* and *Chrng* were previously shown to contribute to nicotine dependence [66], and *Kcnj13* was shown to take part in smooth muscle morphogenesis and trachea development in the mouse respiratory tract [71]. Yin et al. [71] found that mice deficient in *Kcnj13* developed shorter trachea due to loss of function of the potassium channel KCNJ13, which is critical during trachea tubulogenesis. Another candidate region on chromosome 16 is associated with variation in *Deltaproteobacteria* abundance. Within this interval, the core circadian clock gene *Per2* was found near the peak SNP. Interestingly, members of the *Per* subfamily act as tumor suppressor genes in mice, and the downregulation and loss of *PER2* is associated with tumor proliferation and metastasis, including NSCLC [72–74]. In contrast, increased *PER2* expression inhibits cell growth and NSCLC growth [72, 73].

The success of our mapping study relied on our approach to mitigate contamination and the employment of quantitative microbial profiling. Studies comparing ddPCR and qPCR find that while qPCR has a lower detection limit, it yields a worse signal-to-noise ratio when tested with negative controls [56], and yields inconsistent results when tested against intentionally contaminated samples diluted to replicate low-biomass conditions [126]. Further, Taylor et al. [127] note that ddPCR is also particularly advantageous for studies requiring long sample processing times, e.g. where a study cohort might be constructed slowly over time, as the method is robust to batch effects.

In our study, we find that incorporating ddPCR-based quantitative profiling improved the detection of gene-microbe interactions. In particular, it is known that QTL mapping significance thresholds are influenced by the underlying phenotype distribution [128]. Our significance thresholds were assigned based on random and repetitive shuffling of microbial phenotype values across genotypes. The probability of a given genotype/sample to be randomly assigned its original phenotype value is higher when interindividual variation is low, ultimately leading to higher significance thresholds. Notably, our QTL analysis using CMM-QMP phenotypes were assigned lower QTL significance thresholds compared to the QTL analysis using CMM-RA phenotypes (see e.g., *Pelomonas* CMM-RA vs. CMM-QMP QTL on chromosome 11, Fig. 3), as variation within the CMM-QMP is greater than that within the CMM-RA (Additional file 2: Tables S1-2). Thus, it is possible that the increase in interindividual variability among CMM-QMP traits, made possible through ddPCR measurements, better reflects the underlying biological distribution of the trait, which also contributes to lower significance thresholds.

The effectiveness of our mapping approach is congruent with previous employing absolute abundance profiling to characterize resident microbial communities [129–134]. Vandeputte et al. [51] found microbial load to be a key feature of Crohn’s increase disease, with disease being characterized by a reduced bacterial load rather than differences in abundance among disease-associated taxa per se. Similarly, Sibila et al. [135] identified an association between bacterial load and airway inflammation in patients with bronchiectasis. A subsequent clinical trial conducted using the same patient cohort revealed that those with a high bacterial load showed significant improvement after receiving antibiotic therapy in comparison to those with a low bacterial load [135].

## Conclusions

In summary, by combining the unique resource of a high-resolution mouse genetic mapping population together with experimental and computational advances in studying low biomass microbial communities, we demonstrate a novel role for host genetic variation in shaping lung microbiota composition. We find several promising associations for the commonly identified lung taxa *Lactobacillus* and *Pelomonas*. Given that mouse models of human lung diseases, including asthma, chronic obstructive pulmonary disease, and pulmonary fibrosis, have served as powerful tools for understanding pathophysiology and identifying new drug targets [136], our results suggest that the functional relevance of these taxa that may be exploited for future preventative/therapeutic purposes. These approaches outlined here may find useful application in future experimental models of host-microbe interactions in the lung.

## Methods

### Animal husbandry

The analysis of G_15_ intercross mice was approved by the “Ministerium für Energiewende, Landwirtschaft, Umwelt und ländliche Räume des Landes Schleswig- Holstein” in Kiel, Germany (reference number: V 312– 72,241. 122–5 (12 − 2/09)). The G_15_ AIL mouse population was generated by intercrossing four strains, MRL/MpJ, NZM2410/J, BXD2/TyJ, and CAST/EiJ, with equal sex and strain distributions for 15 generations as previously described [27, 42]. The analysis of *Il10* KO and wildtype C57BL/6 mice was performed according to approved animal protocols and institutional guidelines of the Max Planck Institute for Evolutionary Biology in Plön. Mice were maintained and handled in accordance with FELASA guidelines and German animal welfare law (Tierschutzgesetz § 11, permit from Veterinäramt Kreis Plön: 1401–144/PLÖ–004697).

*Il10* KO and wildtype C57BL/6 mice (Jackson Laboratories, Maine, USA) were mated at age of 8–10 weeks to produce F_1_ mice, using both directions of the cross to reduce potential “grandmother,” or legacy effects [137, 138]. Heterozygous F_1_ mice were mated within each cross at 10 weeks and included 11 pairs from each cross. F_2_ mice were weaned at 3 weeks; males and females were housed in separate cages according to family, with mixed genotypes. Mice were maintained in individual ventilated cages (IVCs), type II long (Tecniplast®, Greenline) in a specific pathogen-free facility (MPI für Evolutionsbiologie, Plön, Germany) with a 12-h light/dark cycle. Decalcified water and food (1324, fortified, from Altromin) were provided *ad libidum*. An average of ten mice (range: 9–15) were selected from each sex of each genotype from each cross for tissue extraction at 17 weeks of age.

### Sampling and nucleic acid extraction

The entire lower respiratory tract was dissected and preserved in RNALater (Thermo Fisher Scientific) at 4 °C overnight. Samples were stored at -20 °C after removing RNALater. Approximately the bottom half of the left lobe was obtained for nucleic acid extraction, using tools sterilized with 70% ethanol, RNase-Away (Thermo Fisher Scientific), and sterilizing beads (Fine Science Tools) heated to at least 120 °C. Lung tissues were first homogenized using Lysing Matrix E (MP Biomedicals) and nucleic acid extraction was conducted using the AllPrep 96 DNA/RNA kit (QIAGEN) with on-column DNase I treatment (QIAGEN), according to manufacturer’s protocol, with the exception of TCEP instead of β-mercaptoethanol for the lysis step. A total of 40µL of RNA was eluted by adding 20µL of RNase-free water twice; 30µL of EB buffer was added twice for DNA elution. Concentrations were measured using NanoDrop 1000 (Thermo Fisher Scientific), with RNA samples diluted to equal concentrations (200ng/µL). Reverse transcription was performed according to the manufacturer’s instructions using High-Capacity cDNA Reverse Transcription Kit (Applied Biosystems) using 10µL of template RNA.

### 16S rRNA gene amplicon sequencing

The V1-V2 hypervariable region of the 16S rRNA gene was amplified using primers 27 F and 338R, barcoded with unique eight-based MIDs (multiplex identifiers), using a dual indexing approach on Illumina MiSeq platform for both cDNA and DNA template [27]. We chose the V1-V2 region of the 16S rRNA gene for multiple reasons. In particular, we made previous experience of this primer pair performing well in an earlier low biomass study of the same G_15_ mice [27], which also enables future cross-body site analyses. Three negative extraction controls (NEC) from each extraction plate and one microbial community DNA standard (20ng/µL) (ZymoBIOMICS) were included. Host reads were removed from DNA extracts using “Kneaddata” (v.0.6.1), which removes host reads based on the provided reference database, *Mus musculus*. However, the sequence qualities and read counts were not sufficient after this procedure, and we thus did not further proceed with these sequences generated from the extracted DNA (Additional file 4: Table S8). Sequences were processed using the “DADA2” package (v.1.14.1) [139] with the Ribosomal Database Project (RDP) training set 16 [140] for taxonomic classification, resulting in abundance tables of amplicon sequence variants (ASVs). Results were merged with metadata including sex, age, weight, and cage information, using “phyloseq” (v.1.30.0) [141] for further analysis in R (v.3.6.3).

### ddPCR

A 20µL ddPCR master mix was prepared with QX200™ ddPCR™ EvaGreen^®^ SuperMix (BioRad) following the manufacturer’s instructions (BioRad), with a final primer concentration of 120nM and with 10ng of nucleic acid template. PCR was performed on Bio-Rad C1000 Touch Thermal Cycler with the following conditions: 95 °C for 5 min, 40 cycles at 95 °C for 15 s and 60 °C for 1 min, 4 °C for 5 min, 90 °C for 5 min, and incubation at 10 °C. Final products were transferred to QX200™ Droplet Reader and quantified as gene copies (per 20µL) using Bio-Rad QuantaSoft (v.1.7.4.0917). *Lactobacillus* and *Pelomonas* loads were quantified using genus specific primers on 242 DNA and cDNA samples each (Table 3).

**Table 3:** List of primers used for ddPCR.

	Primer	5’ – 3’	Reference
Total bacteria	63 F	GCAGGCCTAACACATGCAAGTC	[56]
	355R	CTGCTGCCTCCCGTAGGAGT	
*Lactobacillus*	F-Lacto	GAGGCAGCAGTAGGGAATCTTC	[57]
	R-Lacto	GGCCAGTTACTACCTCTATCCTTCTTC	
*Pelomonas*	357 F	CGGGTTGTAAACCGCTTTTGT	
	550R	CGGGGATTTCACCTCTGTCT	

### Contamination assessment and defining core measurable microbiota

To assess for potential contamination, total bacterial load estimates were measured by ddPCR, which together with negative extraction controls (n = 3, i.e., one per 96 well extraction plate) were used for the “frequency” method in the R package “decontam” (v.1.6.0) with a threshold of 0.1. This method identifies contaminants using a de novo classification method based on identifying a negative correlation between concentration (bacterial load) and the frequency of the putative contaminants appearing in samples - the lower the bacterial load, the higher the proportion/frequency of contaminant taxa is expected [34, 38]. ASVs identified as likely contaminants, including those belonging to *Halomonas* and *Shewanella*, were removed from the dataset prior to further analysis. After the decontamination process, samples were rarefied to even sampling depth of 4,000 reads per sample. CMM thresholds are study-specific and reflect the design, body site, and the aims of the study [142]. Here, using the cDNA template samples (RNA-level), we defined the CMM of resident lung microbiota using a 25% prevalence threshold together with a minimum relative abundance threshold of 1%. We reasoned that a 25% prevalence threshold cut-off was appropriate, as taxa below 20% prevalence can be limited in their statistical testability [143, 144] and because prevalence thresholds above 30% may be unnecessarily stringent for statistical reliability [144]. The final CMM among 242 samples included 13 ASVs and 58 taxa from the genus- to phylum-level (CMM-RA).

### Quantitative microbiome profiling

We used ddPCR-based total bacterial load (RNA level) estimates for quantitative microbiome profiling (QMP), whereby 16S rRNA gene relative abundances were corrected using bacterial load measurements and transformed to “absolute” or quantitative abundances. For this, we used R function *rarefy_even_sampling_depth* (https://github.com/raeslab/QMP) [51], which rarefies samples to even sampling depth, defined as the ratio between sequencing depth and bacterial load (here, based on ddPCR). Among the QMP dataset (CMM-QMP), taxa and ASVs included in the CMM were further selected for mapping.

### QTL mapping

Prior to mapping, summary statistics were performed on all traits including CMM-RA, CMM-QMP, and ddPCR-DNA/RNA in R studio (v.1.2.1335) with R (v.3.6.3) (Additional file 2: Tables S1-3). In order to perform a log_10_ transformation of relative abundance values, a value of 0.5 was added to the absolute abundances of all CMMs prior to converting the absolute abundances into relative abundances. Then, linear mixed effects analysis was performed on these traits using “lme4” (v.1.1–10) [145]. Variance was estimated using “r.squaredGLMM” in “MuMIn” (v.1.43.6) [146] and “VarCorr” in “lme4” for fixed and random effects, respectively.

Linkage mapping was performed using “DOQTL” (1.6.0) [147] and “QTLRel” (0.2.14) [148] in R (v3.2.2), whereby we fit an additive model that regresses the log_10_-transformed traits on the four founder haplotype contributions. Genotype data were previously collected and described in Belheouane et al. [27]. Briefly, the data was obtained by extracting DNA from liver tissue, which was processed using the MegaMuga (Illumina) array to obtain host genotypes. A kinship matrix was defined using “kinship.probs” in “DOQTL” to adjust for the kinship between animals [27]. A 3D-array of founder haplotype contributions (sample # x 4 founders x marker #) [27] and kinship matrix, along with sex and age as fixed variables and cage information as random variable, were included for linkage mapping.

Permutations were run in R (v.3.2.2) for each trait by shuffling the phenotypic data to define significance thresholds at both the 90th and 95th percentiles of LOD scores [27]. Permutations were run 10,000 times, a ten-fold increase from minimum recommendations by Gatti et al. [147]. QTL confidence intervals were defined at 1.5 LOD score drops on either side of the QTL peak. After QTL mapping, genes located within confidence intervals were examined and then plotted using “get.mgi.features” and “gene.plot” in “DOQTL” to identify potential candidate genes.

## Electronic supplementary material

Below is the link to the electronic supplementary material.

**Additional file 1: Figure S1.** Lung bacterial community composition of AIL mice at ASV level.

**Additional file 2: Table S1-3.** Summary statistics of CMM-RA, CMM-QMP, and ddPCR-DNA/RNA traits, respectively. **Table S4-6.** Sources of variation for CMM-RA, CMM-QMP, and ddPCR-DNA/RNA traits, respectively.

**Additional file 3: Table S7.** QTL mapping statistics for CMM-RA, CMM-QMP, and ddPCR-DNA/RNA with significant and suggestive associations.

**Additional file 4: Table S8.** Average number of reads from each step of the DADA2 pipeline.

## Data Availability

Sequencing data generated and analyzed is available in the NCBI Sequence Read Archive (SRA) under BioProject PRJNA856849 (https://dataview.ncbi.nlm.nih.gov/object/PRJNA856849?reviewer=3ko45p5hm7jq0b3bkp2vstedtc). Scripts used for the mapping as well as raw genotype and meta data are available at https://github.com/ceciliajchung/G15_lung.

## Abbreviations

AIL: Advanced intercross line
ASV: Amplicon sequence variant
BAL: Bronchoalveolar lavage
CMM: Core measurable microbiota
ddPCR: Droplet digital PCR
LOD: Logarithm of odds
PCR: Polymerase chain reaction
QMP: Quantitative microbiome profiling
qPCR: quantitative PCR
QTL: Quantitative trait loci
RA: Relative abundance.
