# Supplementary figures and images for: Genome-wide mapping of gene-microbe interactions in the murine lung microbiota based on quantitative microbial profiling
Source: Anim Microbiome. 2023 Jun 1;5:31. doi: 10.1186/s42523-023-00250-y (PMC10234247; doi:10.1186/s42523-023-00250-y)

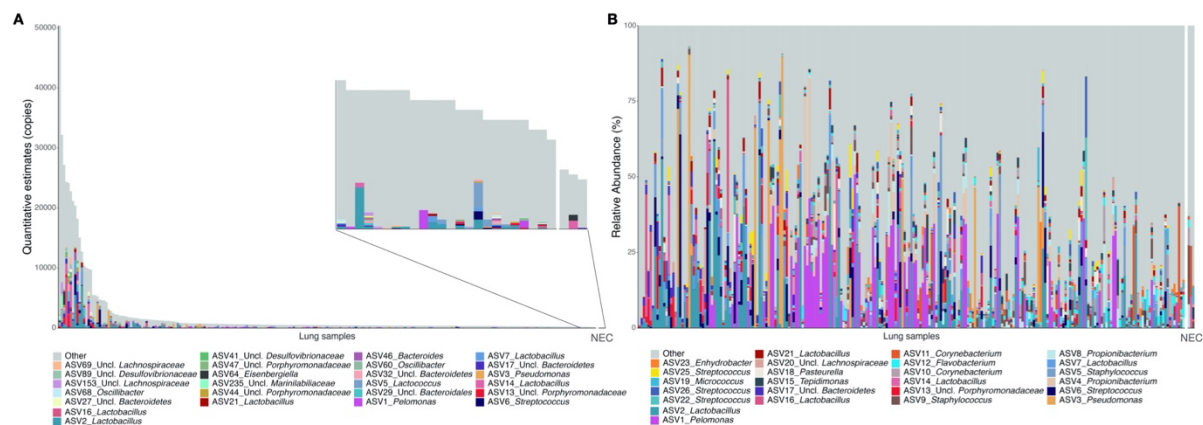

Supplement: Supplementary file 1 — Additional file 1: Figure S1. Lung bacterial community composition of AIL mice at ASV level. [file 42523_2023_250_MOESM1_ESM.pdf]
